# Supplementary material for: Optimization of 3D Printing Parameters of High Viscosity PEEK/30GF Composites
Source: Polymers (Basel). 2024 Sep 14;16(18):2601. doi: 10.3390/polym16182601 (PMC11435271; doi:10.3390/polym16182601)
Supplement: Supplementary file 1 [file polymers-16-02601-s001.zip › polymers-3153056-supplementary.pdf]

Table S1. Parameters of 3D-printing (input parameters)

The number of experiment	Extruder temperature, °C (nozzle temperature)	Travel (print) speed, mm/s	Extrusion rate, revolutions/minute (rev/min)
1	420	20	4
2	420	30	4.5
3	420	40	5
4	440	20	4.5
5	440	30	5
6	440	40	4
7	460	20	5
8	460	30	4
9	460	40	4.5
10	460	20	4
11	380	20	5
12	380	40	5
13	400	30	5
14	400	10	5
15	440	30	3.5
16a	340	20	4
16b	340	30	4
16c	340	40	4
16d	340	20	4.5
16e	340	30	4.5
16f	340	40	4.5
16g	340	20	5
16h	340	30	5
16i	340	40	5
17a	560	20	4
17b	560	30	4
17c	560	40	4
17d	560	20	4.5
17e	560	30	4.5
17f	560	40	4.5
17g	560	20	5
17h	560	30	5
17i	560	40	5
18a	380	0	4
18b	380	0	4.5
18c	380	0	5
18d	420	0	4
18e	420	0	4.5
18f	420	0	5
18g	460	0	4

18h	460	0	4.5
18i	460	0	5
19a	380	60	4
19b	420	60	4
19c	460	60	4
19d	380	60	4.5
19e	420	60	4.5
19f	460	60	4.5
19g	380	60	5
19h	420	60	5
19i	460	60	5
20a	380	20	0
20b	420	30	0
20c	460	40	0
20d	380	20	0
20e	420	30	0
20f	460	40	0
20g	380	20	0
20h	420	30	0
20i	460	40	0
21a	380	20	8
21b	420	30	8
21c	460	40	8
21d	380	20	8
21e	420	30	8
21f	460	40	8
21g	380	20	8
21h	420	30	8
21i	460	40	8
**The range under analysis**			
Min	340	0	0
Max	560	60	8

Table S2. Experimentally measured properties (output parameters)

The number of experiment	Tensile strength, MPa	Elastic modulus, MPa	Elongation at break, %
101	96.4	1851	5.36
102	88	1917	4.49
103	85.5	2200	4.47
104	74.44	2430	4.94
105	82.8	2196	4.69
106	81.3	2029	4.51
107	86.8	2716	4.91
108	101.37	2891	4.68
109	89.4	2153	4.69
110	79.3	2726	4.06

111	83.2	2082	5.21
112	83.94	2436	5.18
113	94.1	2330	5.43
114	80.3	2338	3.93
115	87.7	2707	4.22
116	102.03	2447	5.24
117	95.4	2306	5.09
118	90.1	2194	4.66
119	90.2	2251	5.58
120	102.45	2683	5.09
121	85.4	2774	4.48
122	80.5	2412	4.74
123	84.4	1949	4.72
124	88.27	2167	4.29
125	97.2	2278	5.22
126	93.8	2596	4.94
127	92.1	2793	4.64
128	94.83	2772	4.36
129	93.5	2552	4.67
130	99.3	2547	6.05
131	90	2544	4.99
132	92.37	2526	4.99
133	87.3	2733	4.73
134	82.2	2181	4.3
135	85.5	1861	4.31
136	84.84	2737	3.74
4	80.6	2835	-
11	100.6	2463	4.6
17	78.9	2503	4.2
23	75.4	2588	3.8
36	92.7	3146	4.1
38	102.3	1714	4.7
30	102.7	2754	5.2
5	73.2	1807	4.1
6	90.7	2928	5.2
12	81.7	1848	4.4
18	79.5	1723	4.7
24	85.1	2053	5
31	86.5	1690	4.7
7	72.3	1878	4.3
13	82.2	1840	5.2
19	75.1	2192	4.1
25	72.4	2567	5
32	96.1	2615	4.8
41	74.3	3269	2.9
8	84.5	2983	4

14	85.7	2463	4.9
20	80.3	2234	4.8
26	86.5	2363	5
33	85.1	2595	4
9	78	1949	4.5
15	-	-	-
21	-	-	-
27	82.6	2559	4.5
28	100	3339	4.5
34	89.9	3264	4.4
40	98.3	2391	5
42	76.3	2381	4.1
10	63.7	2436	3.5
16	67.9	2294	3.8
22	78	2121	3.8
29	81.1	2048	3.6
35	71.6	2208	3.4
37	60.3	2745	2.7
39	45.1	2442	2.3

Apriory knowledge

The number of experiment	Tensile strength, MPa	Elastic modulus, MPa	Elongation at break, %
16a	0	0	0
16b	0	0	0
16c	0	0	0
16d	0	0	0
16e	0	0	0
16f	0	0	0
16g	0	0	0
16h	0	0	0
16i	0	0	0
17a	45	1500	2
17b	45	1500	2
17c	45	1500	2
17d	45	1500	2
17e	45	1500	2
17f	45	1500	2
17g	45	1500	2
17h	45	1500	2
17i	45	1500	2
18a	0	0	0
18b	0	0	0
18c	0	0	0
18d	0	0	0
18e	0	0	0
18f	0	0	0

18g	0	0	0
18h	0	0	0
18i	0	0	0
19a	50	1700	2.5
19b	50	1700	2.5
19c	50	1700	2.5
19d	50	1700	2.5
19e	50	1700	2.5
19f	50	1700	2.5
19g	50	1700	2.5
19h	50	1700	2.5
19i	50	1700	2.5
20a	0	0	0
20b	0	0	0
20c	0	0	0
20d	0	0	0
20e	0	0	0
20f	0	0	0
20g	0	0	0
20h	0	0	0
20i	0	0	0
21a	60	1800	3
21b	60	1800	3
21c	60	1800	3
21d	60	1800	3
21e	60	1800	3
21f	60	1800	3
21g	60	1800	3
21h	60	1800	3
21i	60	1800	3

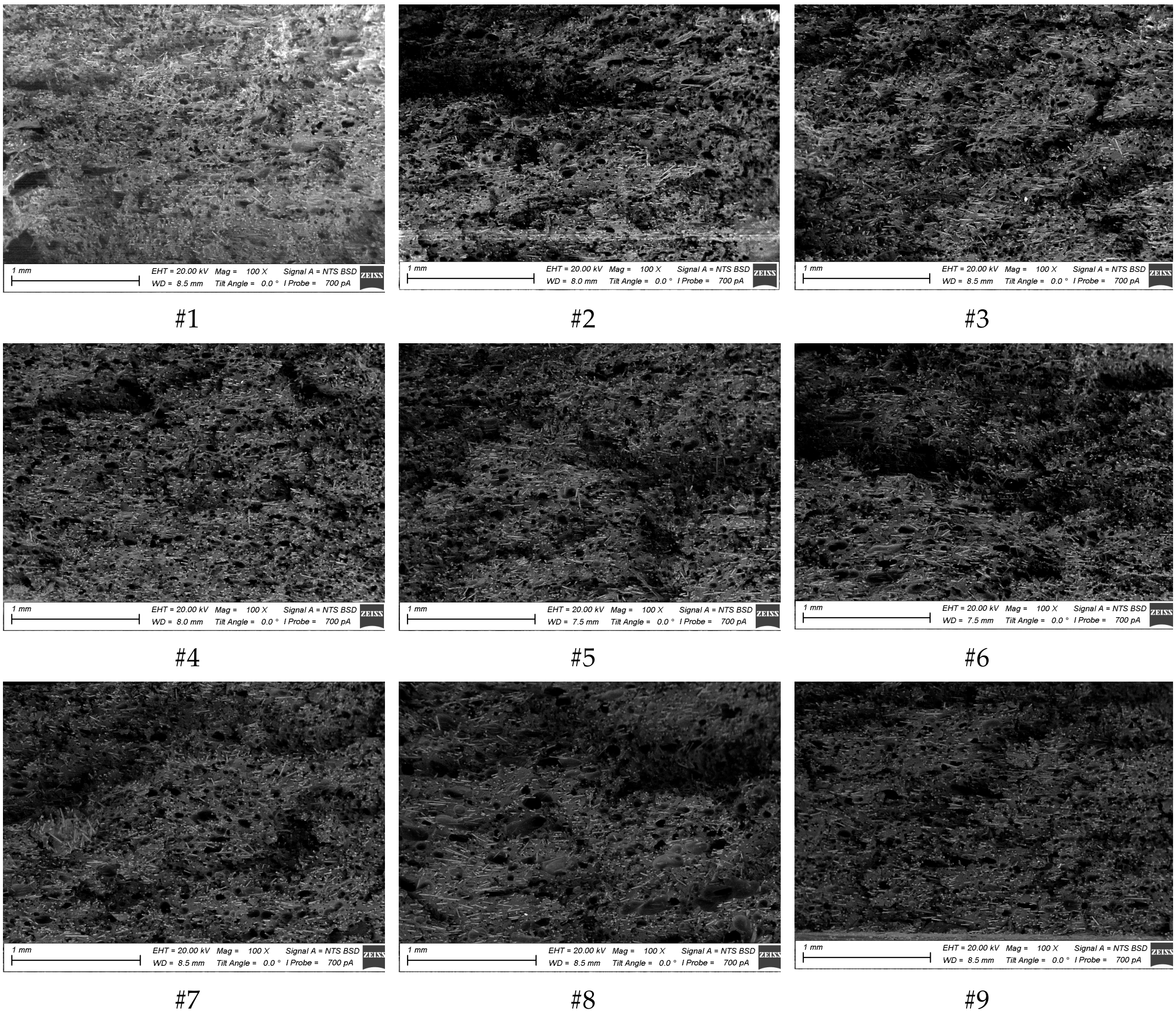

Figure S1. The lower magnification SEM micrographs of the PEEK/30GF composites additively manufactured using the modes presented in Table 2.

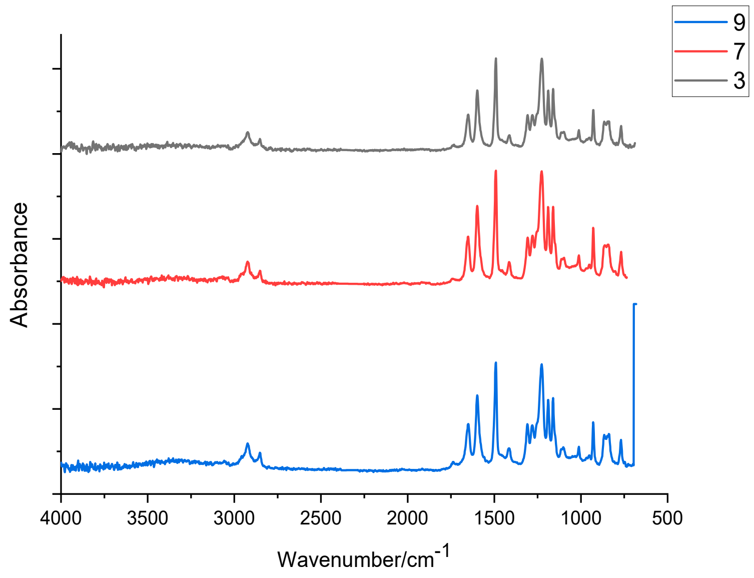

Figure S2. The IR spectra of the samples of the PEEK/30GF composite additively manufactured using modes 3, 7 and 9, according to Table 2.
